# Retraction: Investigation of yellow horn (*Xanthoceras sorbifolia* Bunge) transcriptome in response to different abiotic stresses: a comparative RNA-Seq study

**DOI:** 10.1039/d0ra90119a

**Published:** 2020-11-26

**Authors:** Yanhe Lang, Zhi Liu, Zhimin Zheng

**Affiliations:** State Key Laboratory of Tree Genetics and Breeding Laboratory, Ministry of Education, Alkali Soil Natural Environmental Science Center (ASNESC), College of Life Science, Northeast Forestry University Harbin Heilongjiang Province China langyanhe@163.com +86-151-0453-8096

## Abstract

Retraction of ‘Investigation of yellow horn (*Xanthoceras sorbifolia* Bunge) transcriptome in response to different abiotic stresses: a comparative RNA-Seq study’ by Yanhe Lang *et al.*, *RSC Adv.*, 2020, **10**, 6512–6519, DOI: 10.1039/C9RA09535G.

We, the named authors, hereby wholly retract this *RSC Advances* article due to issues with the experimental design and data analysis, which were discovered after carrying out the follow-up experiments.

(1) In this study, only one control group was set in accordance to both 24 h and 48 h stress treatment. However, as a matter of fact, two control groups should have been set in the context of the two treatment times (24 h and 48 h), respectively. It is assumed that the differentially expressed genes (DEGs) of yellow horn seedings should be identical without stress treatment at both 24 h and 48 h, but in fact there existed some differences. And this led to the incorrect MDS analysis.

(2) Selection of treatment time (24 h and 48 h) was inappropriate. In the follow-up experiments, other treatment times (*i.e.* 0 h, 3 h, 6 h, 9 h and 12 h) were tried, and it was found that after stress treatment for 6 h or 9 h, almost all stress-responsive genes were up-regulated; while after treatment for 24 h and 48 h, most stress-responsive genes were found to be generally down-regulated. So treatment for 6 h and 9 h may be the better choice.

(3) Salt concentration was not appropriate; 200 mM or higher salt concentration would be better, as evidenced by transcriptomic analysis, although this still needs further evaluation *via* large-scale transcriptomic sequencing.

(4) The transcriptomic data was not properly administered, arranged and named in time, due to restrictions due to Covid-19. This led to errors in the subsequent bioinformatics analysis and therefore the conclusions of this paper are not supported. We therefore regretfully wish to retract the results presented in this paper and further improve the experiment. Further detailed studies using the improved methods are forthcoming.

Signed: Yanhe Lang, Zhi Liu and Zhimin Zheng

Date: 16^th^, October, 2020

Retraction endorsed by Laura Fisher, Executive Editor, *RSC Advances*

## Supplementary Material

